# Characterizing somatic mutations in ovarian cancer germline risk regions

**DOI:** 10.1038/s42003-025-08072-1

**Published:** 2025-04-29

**Authors:** Ping-Hung Lai, Jonathan P. Tyrer, Paul Pharoah, Simon A. Gayther, Michelle R. Jones, Pei-Chen Peng

**Affiliations:** 1 Department of Computational Biomedicine, Cedars-Sinai Medical Center, West Hollywood, CA USA; 2 CR-UK Department of Oncology, University of Cambridge, Strangeways Research Laboratory, Cambridge, UK; 3 Center for Inherited Oncogenesis, Department of Medicine, UT Health San Antonio, San Antonio, TX USA; 4 Center for Bioinformatics and Functional Genomics, Department of Biomedical Sciences, Cedars-Sinai Medical Center, Los Angeles, CA USA

**Keywords:** Computational biology and bioinformatics, Cancer genomics, Cancer genetics

## Abstract

Epithelial ovarian cancer (EOC) genetics research has been focused on germline or somatic mutations independently. Emerging evidence suggests that the somatic mutational landscape can be shaped by the germline genetic background. In this study, we aim to unravel the role of somatic alterations within EOC germline susceptibility regions by incorporating functional annotations. We investigate somatic events, including mutational signatures, point mutations, copy number alterations, and transcription factor binding disruptions, within 33 EOC germline susceptibility regions. Our analysis identifies significant associations between candidate germline susceptibility genes and somatic mutational signatures known to be key risk factors for EOC, such as mismatch repair deficiency, age-related mutagenesis, and homologous recombination deficiency. In addition, we find somatic point mutations and copy number alterations are significantly enriched in histotype-specific active enhancers and promoters within EOC risk loci. Furthermore, we examine the impact of germline variants and somatic mutations on transcription factor binding sites, identifying cancer developmental transcription factor motifs frequently affected by both types of mutations. Overall, our study highlights the importance of integrating germline and somatic mutations with regulatory and epigenomic data to gain insights into the genetic basis of EOC.

## Abstract (web summary)

Integrating germline and somatic mutations with regulatory data reveals how epithelial ovarian cancer risk regions shape somatic alterations, highlighting key mutational signatures, enhancer disruptions, and transcription factor binding changes.

## Introduction

Epithelial ovarian cancer (EOC) is one of the most lethal gynecologic diseases and ranks as the eighth most frequently occurring cancer in women worldwide^1,2^. Owing to its vague symptoms, EOCs are often diagnosed at a late stage resulting in a poor overall survival and are notoriously insidious in nature^3^. Early detection is challenging due to limited screening tests^4^. Although surgical tumor removal followed by the platinum chemotherapy is often performed, over 70% of EOC patients experience rapid relapse^5^, accompanied by an accumulation of genomic and epigenomic alterations. Understanding the cell-type specific genetic architecture of EOC is crucial for elucidating cancer risk and related outcomes.

Inherited germline variants and acquired somatic mutations play pivotal roles in defining the genetic basis of cancer. Germline variants in high penetrance genes, such as *BRCA1*, are inherited in a Mendelian fashion and confer susceptibility to EOC and other cancer types. More recently, genome-wide association studies (GWASs) have identified numerous common low penetrance alleles associated with EOC risk^6–9^. The majority of germline susceptible regions are located in the noncoding genome. Partitioned SNP heritability across functional annotations has indicated a significant contribution of regulatory elements to EOC heritability^10^. On the other hand, somatic alterations are acquired over time and are sporadic in the genome^11^. Accumulated somatic mutations affecting several genes can disrupt critical biological mechanisms, such as DNA replication and division or DNA damage repair^12,13^, conferring a selective growth advantage for tumor cells. Previous studies have demonstrated that age- and homologous recombination-associated mutational signatures^14,15^ and copy number alterations (CNAs)^14,16,17^ in oncogenes contributed to EOC chemotherapy resistance.

While both germline variants and somatic alterations are crucial to tumor formation, they are often studied separately. Joint analysis of these variants could provide more comprehensive insights, as several lines of evidence suggest an interplay between germline variants and somatic alterations in cancer development^18^. For example, in EOC both germline and different somatic pathogenic mutations in the *BRCA1* gene have been identified; and for breast cancer, there is a significant but inverse association between the germline risk variant rs2588809 in the DNA repair gene *RAD51B* and total somatic mutation count^19^. More recently, Liu et al.^20^ identified 17 statistically significant associations between somatic mutational profiles and germline polygenic risk scores, including the overall breast cancer polygenic risk scores with the somatic mutation counts of APOBEC-related signatures. Kanchi et al.^21^ reported 237 germline truncating or missense variants associated with somatic events, such as co-localization with somatic mutations in the same gene. However, these studies primarily focused on the coding genome, despite the majority of germline and somatic alterations occurring in the noncoding genome^10,22^. Furthermore, the biological and regulatory mechanisms for somatic mutations in germline risk regions remain largely uncharacterized.

**Fig. 1:**
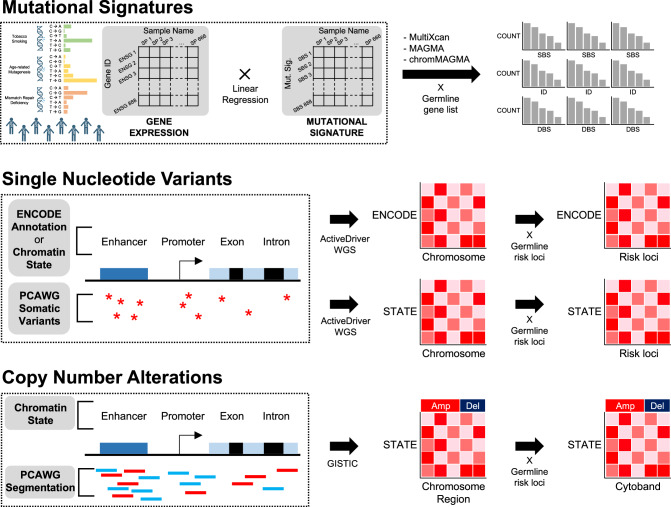
Mutational landscape in the germline and somatic mutations co-localized regions. Different types of somatic alterations were examined with specific analytical tools. Linear regression models were used to explore the relationship between mutational signatures and candidate germline susceptibility genes (top). Enrichment analyses were performed to identify functional annotations significant for somatic point mutations (middle) and copy number alterations (bottom) in the germline risk regions.

In the current study, we examined somatic alterations located within EOC germline susceptible regions and hypothesized that they may function by altering the activity of host regulatory elements that are active in ovarian precursor or cancer cell types. We applied systematic, computational approaches to study the regulatory and epigenetic landscape of somatic mutations in EOC germline risk regions (Fig. 1). First, we began by examining the association between germline susceptibility genes and somatic mutational signatures. Next, we investigated somatic mutational events including point mutation, CNAs, and interruptions of transcription factor (TF) bindings, in 33 EOC common low-penetrance germline genetic risk regions. This comprehensive approach provided a deeper understanding of the properties of somatic alterations in EOC germline risk regions, shedding light on potential mechanisms driving tumorigenesis and disease progression.

## Results

### Somatic mutational signatures are significantly associated with germline susceptibility genes in ovarian cancer

**Fig. 2:**
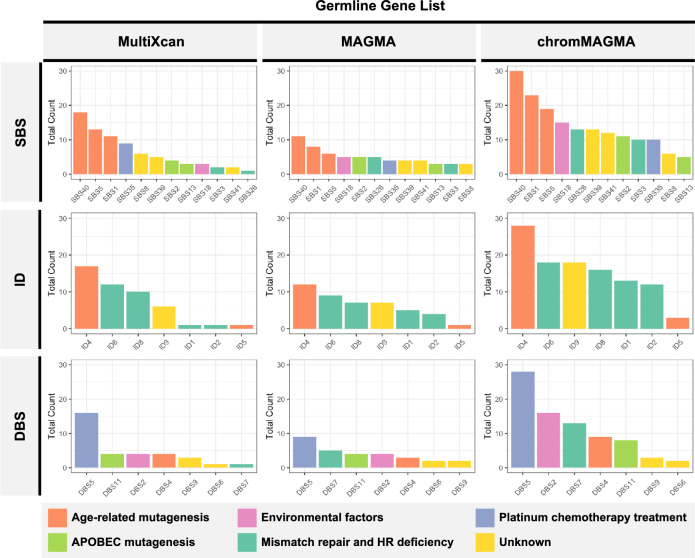
Counts of genes significantly correlated to the COSMIC mutational signatures. Rows represent COSMIC mutational signatures, featuring single base substitutions (SBS), small insertions and deletions (ID), and double base substitutions (DBS). Columns display bar charts of counts for significantly correlated germline susceptibility genes identified from MultiXcan, MAGMA, and chromMAGMA. Proposed etiologies of the significantly correlated mutational signatures are categorized in the bottom.

Somatic mutations can be characterized by several recognizable patterns known as mutational signatures^23^. We employed linear regression models to elucidate the relationship between mutational signatures and germline susceptibility genes. We focused on medium and low-penetrance susceptible genes identified by three different approaches: MultiXcan (*n* = 83 genes)^24^, MAGMA (*n* = 68 genes)^25^, and chromMAGMA (*n* = 155 genes)^26^. (Supplementary Data 1). Gene expression data from RNA-seq of 89 ovarian samples from the pan-cancer analysis of whole genomes (PCAWG) consortium and its corresponding mutational signatures from the Catalog Of Somatic Mutations In Cancer (COSMIC) database were utilized to find significant associations between germline susceptibility genes and mutational signatures. Among three gene lists, we identified on average 54 germline susceptibility genes significantly associated with 12 single base substitutions (SBS) mutational signatures, 39 genes associated with small insertions and deletions (ID) signatures, and 40 genes associated with doublet base substitutions (DBS) signatures (Fig. 2 and Supplementary Data 2). In random permutations analysis, the results demonstrate that chromMAGMA predicted germline susceptible genes are significantly more likely to be associated with mutational signatures (*p* = 0.0003 for SBS, *p* = 0.0019 for ID, and *p* = 0.0699 for DBS), compared to the genes identified from MultiXcan and MAGMA (Supplementary Fig. 1). We note that there is not yet evidence that individual germline variants are related to mutational signatures; rather, our findings indicate that the expression of genes identified in GWAS are correlated with mutational signatures in EOC samples.

The identified mutational signatures can be categorized into six groups, based on the proposed etiology in COSMIC database^23^: mismatch repair deficiency (SBS26 and, DBS7), homologous recombination deficiency (SBS3, ID1, ID2, ID6, and ID8), age-related mutagenesis (SBS40, SBS5, SBS1, ID4, ID5, and DBS4), platinum chemotherapy treatment (SBS35 and DBS5), environmental factors (SBS18 and DBS2), and APOBEC mutagenesis (SBS2, SBS13, and DBS11), all of which are known to be key risk factors for ovarian cancer. Among these, the most frequently associated susceptibility genes include *ACOXL*, *TBKBP1*, *HOXD11*, and *CPAMD8*, each linked to 11, 11, nine, and nine mutational signatures, respectively. Importantly, *TBKBP1* has been implicated in mediating both tumor growth and immunosuppression^27^, while *HOX* genes have shown highly dysregulated expression in ovarian cancers of all histotypes^28^. The most significant associations are *BEST1* with DBS5 (*p* = 6.45E-18), *HOXD10* with ID1 (*p* = 6.76E-17), and *HOXD10* with ID2 (*p* = 6.77E-17).

### Somatic point mutations are significantly enriched in active regulatory elements in ovarian cancer risk loci

**Fig. 3:**
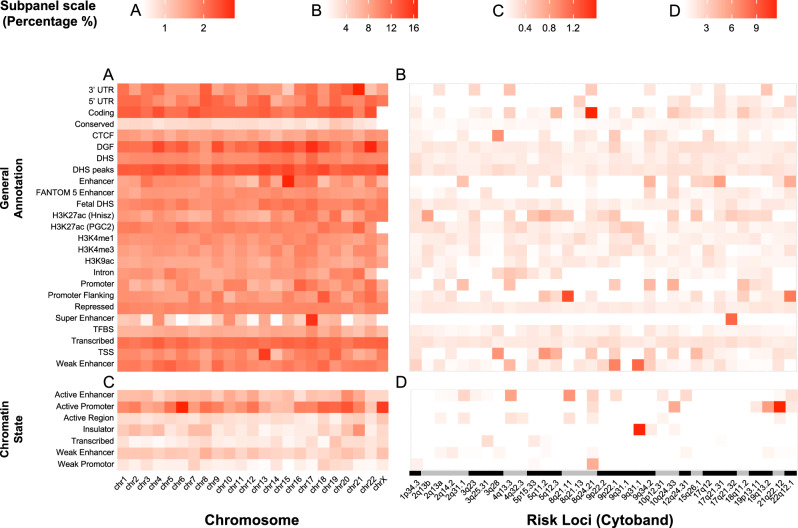
Enrichment of frequently somatic mutated regions. Enrichment analyses (**A**) across cell-type agnostic annotations, (**B**) across cell-type agnostic annotations, within 33 EOC germline risk loci, (**C**) across EOC related chromatin states, and (**D**) across EOC related chromatin states, within 33 EOC germline risk loci.

Since the majority of somatic point mutations are in the noncoding genome, we next asked if somatic mutations in regions frequently mutated in ovarian cancers (1) are located in the regulatory regions; and (2) coincide with regions containing germline alleles for ovarian cancer. We used ActiveDriverWGS to identify genomic regions frequently mutated by somatic mutations and characterized them with functional annotations. We began the enrichment analysis with 25 functional annotations agnostic to cell-type^29^ and found active regions (DNase I Hypersensitive Sites (DHS) peaks), coding regions, and transcribed regions that are frequently mutated genome-wide. Furthermore, we observed that 2.79% of 3′UTRs on chromosome 21 and 2.76% of enhancers on chromosome 15 are significantly mutated (Fig. 3A). Subsequently, we focused on 33 independent, fine mapped ovarian cancer risk loci^9^ and performed an enrichment analysis (Fig. 3B). Unlike the observations from our genome-wide enrichment analyses, there is no single annotation that is enriched across all risk loci. Together, enhancers, promoter flanking regions, and active H3K27ac marks show enrichment in most loci. Specifically, at 8q24.21, there was enrichment (16.67%) for the coding regions that are significantly mutated; 14.29% of promoter flanking regions at 8q21.11 that are significantly mutated; and 12.5% of super enhancers at 17q21.32 that are significantly mutated.

Based on the significant enrichment observed in cell type agnostic regulatory annotations, we further characterized the frequently mutated regions by seven ovarian cancer related chromatin states. The chromatin states, derived from 18 EOC precursor or EOC related cell lines, were annotated for active region, active and weak promoters, active and weak enhancers, insulators, and transcribed regions^9^. Active promoters and active enhancers have a higher percentage of regions mutated by somatic mutations (Fig. 3C). The most pronounced enrichment was found in ovarian cancer-related active promoters on chromosome 6. Once again, the regions were limited to ovarian cancer risk loci, where we found significant enrichments in active promoters and active enhancers across most of the ovarian cancer risk loci (Fig. 3D). Of note 11.36% of ovarian cancer-related active promoters at 21q22.12 were significantly mutated, and 11.11% of ovarian cancer-related insulators at 9q31.1 were significantly mutated (Supplementary Data 3 and 4).

### Somatic CNAs are enriched in ovarian cancer-associated enhancers

**Fig. 4:**
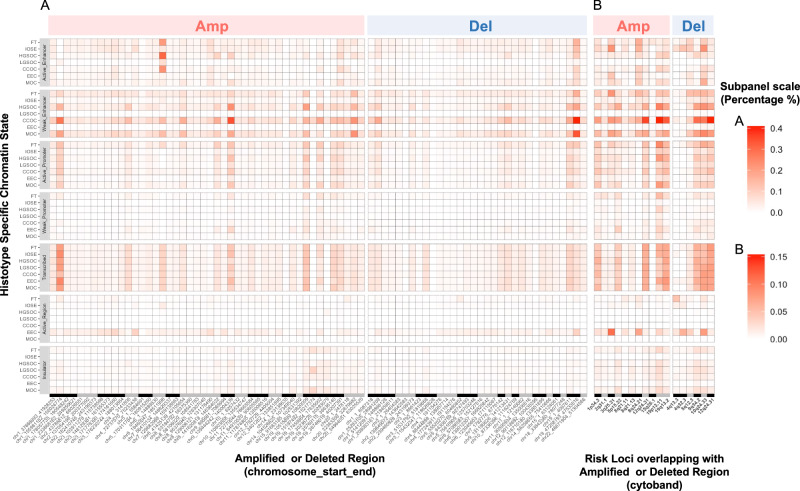
Enrichment of significantly amplified or deleted copy number alterations. **A** Significant copy number alterations within EOC histotype-related chromatin states. HGSOC high-grade serous ovarian cancer, LGSOC low-grade serous ovarian cancer, CCOC clear cell ovarian cancer, EEC endometriosis epithelial cells, MOC mucinous ovarian cancer, FT fallopian tube epithelial cells, and IOSE ovarian surface epithelial cells. **B** Significant copy number alterations overlapped with EOC germline risk loci.

CNAs in the genome are substantial drivers of ovarian cancer development^14^. We explored the epigenetic landscape of somatic CNAs both genome-wide and specifically at ovarian cancer germline genetic risk loci. Using GISTIC^30^, we identified 46 significantly amplified and 32 significantly deleted regions from PCAWG segmentation data analysis of ovarian tumors (Supplementary Data 5). The most significant amplification was observed at 8q24.21 (*q*-value = 2.01E-37). Both chromosome 8 and chromosome 19 have the highest number of CNAs, with six out of 46 identified. Histotype specific chromatin states in seven normal ovarian precursor cell types or ovarian cancer-associated cell lines for different subtypes were used to annotate the significant somatic CNAs. We observed that weak enhancers and transcribed regions are more overlapped with somatic CNAs (Fig. 4A) than any other chromatin states. Notably, there was a higher percentage of CNAs identified in weak enhancers in the high-grade serous ovarian cancer (HGSOC) and clear cell ovarian cancer (CCOC) histotypes across the entire genome, with a higher frequency for amplifications on chromosomes 8 and 19 and deletions on chromosome 1.

We then intersected the significantly amplified or deleted regions with the ovarian cancer risk loci, and examined the distribution of those variations (Fig. 4B, Supplementary Data 6). Among 33 risk loci, 16 have CNAs, with more amplification (*n* = 11) than deletions (*n* = 6). Transcribed regions have universal characteristics among all histotypes, while active enhancers, weak enhancers, and active promoters exhibit histotype-dependent properties. Specifically, more CNAs were found in weak enhancers in HGSOC, CCOC, and MOC.

### TFBSs related to cancer development are frequently disrupted

Dysregulation of TF binding sites (TFBSs) can lead to aberrant gene expression patterns, contributing to carcinogenesis and tumor progression. We, therefore, investigated TFBSs frequently impacted by germline variants and somatic mutations within regulatory regions of the genome. Our focus was on 32 regulatory elements with the significantly high frequency of somatic mutations identified above (Fig. 3B, D). We first examined TFBSs significantly disrupted by 4008 credible causal germline variants associated with EOC identified in our previous study^9^, using the statistical tool MotifBreakR. Among the 746 human TFs we investigated, the binding sites of 202 unique TFs have been significantly disrupted by EOC credible causal germline variants (Supplementary Data 7). The most frequently disrupted TF motifs were for REST, disrupted by eight germline variants; TP53, disrupted by seven germline variants; STAT1, which was disturbed by five germline variants; and MAFK, broken by five germline variants (Supplementary Data 7). For somatic mutations, we applied ActiveDriverWGS to identify TFBSs that are highly mutated in regulatory elements. The TFBSs mostly disrupted by somatic mutations were ZNF354C, KLF9, IRF7, and IRF9, each impacted by four somatic mutations. These findings are based on ActiveDriverWGS results filtered by a minimum of three observed mutations in the regulatory element (Supplementary Data 8). Finally, we intersected the two lists of frequently interrupted TFBSs and found 23 TFs in common (Table 1). These TFs have been reported to be involved in cancer development and differentiation (EGR3, ELF3, ETV2, FOXL1^31^, HOXC11, HOXC12, HOXD12, PPARA::RXRA, SOX10, SP2, SP4, SP8, TBX15, and TWIST1), immune response and modulation (HSF2, IRF1, IRF2, IRF7, IRF9^32^, and SPI1), and metastasis and invasion (FOXP3^33^, KLF9, and RORB).

**Table 1:** TFs that are frequently disrupted by germline risk variants and somatic mutations

Functions	TF
Cancer development and differentiation	ETV2
	FOXL1
	HOXC11, HOXC12, and HOXD12
	SOX10
	TBX15
	TWIST1
	RORB
	SP2, SP4, and SP8
Immune response and modulation	EGR3
	ELF3
	FOXP3
	IRF1, IRF2, IRF7, and IRF9
	SPI1
	HSF2
Metabolism and invasion	PPARA::RXRA
	KLF9

## Discussion

Characterizing the regulatory landscape of somatic alterations in the germline risk regions is an important step in delineating the biological mechanisms underlying disease. This study utilized genetics and transcriptomic data from ovarian cancer samples to reveal significant associations between candidate germline susceptibility genes and somatic mutational signatures. We also explored somatic point mutations and somatic CNAs and their enrichment within general functional annotations and ovarian cancer-associated chromatin states. The findings suggested that histotype-specific active enhancers and promoters in EOC risk loci exhibit a higher percentage of somatic mutations and CNAs. Furthermore, we examined the disruption of TFBSs by germline variants and somatic mutations, identifying cancer developmental TF motifs frequently impacted by both types of mutations. These findings shed light on the somatic influences on the EOC germline risk regions and provide a functional roadmap for further research and therapeutic interventions.

Somatic mutations emerge on the background of germline variants^18^, providing a selective advantage to cells during tumorigenesis. In this study, we found that age-related, DNA damage repairs, and APOBEC mutational signatures are significantly associated with medium/low-penetrance germline susceptible genes in EOC. The findings align with previous studies that have conducted similar genome-wide analyses by associating somatic mutational signatures with common germline variants^34^ or germline polygenic scores^20^ at the pan-cancer level. Specifically, we identified APOBEC mutagenesis (SBS2, SBS13, and DBS11) and age-related mutagenesis (SBS40, SBS5, SBS1, ID4, ID5, and DBS4) significantly associated with germline susceptibility genes, including *TBKBP1* and *HOX* genes. Notably, our analyses expanded to DBS and ID signatures, in addition to SBS, making this the first study to focus specifically on EOC in this context.

Identifying the functional effects on germline variants and somatic point mutations is crucial in delineating the biological mechanisms underlying disease. Since regulatory elements are largely tissues and tumor-type specific, detailed profiling of ovarian cancer chromatin states is essential. We identified recurrent somatic point mutations in cell-type specific active promoters and enhancers that coincide in ovarian cancer risk loci. Previously, the PCAWG consortium had characterized recurrent somatic point mutation in the noncoding genome but with fewer genomic annotations and no epigenetic data^22^. Our approach confirmed the presence of these driver mutations in four genes (*CDK12*, *LATS1*, *RB1*, and *TP53*) and seven genomic regions reported by PCAWG in the ovarian cancer dataset (Supplementary Data 9), validating the use of our functional annotations and chromatin states. Furthermore, our results revealed that epigenomic enrichment profiles differed from locus to locus, suggesting these loci are engaged in distinctive modes of regulation. These findings align with reports by us and others^10,35–38^ that each ovarian cancer susceptibility locus is mediated by unique regulatory elements altering gene expression levels. Additionally, we identified overlaps in four regions from our study with those reported in a recent study^39^, which identified over 100 breast and ovarian cancer susceptibility genes through cis and trans-eQTL transcriptome-wide association studies (TWASs). The shared regions, including *RP11-259G18.1* (17:46267037), *PLEKHM1* (17:45435900), *KANSL1-AS1* (17:46193576), and *LINC00886* (3:156747346), suggest common biological pathways and mechanisms, further strengthening the relevance of our findings to ovarian cancer susceptibility.

We acknowledge that while we followed our previous works^9,40^ and other studies^41–43^ to define the germline risk region as ±500 kb flanking the index SNP, extending the flanking region to 1 Mb or 1 cM could potentially uncover additional insights. Furthermore, while human primary tissue specimens better represent tumors than immortalized normal and cancer cell lines used in this study^10^, the publicly available EOC histone marks data from cell lines provide a broader range of chromatin states, enabling more comprehensive annotation of noncoding regions, such as enhancers and promoters. Future efforts could focus on stratifying tumor samples according to inherited polygenic risk to discover recurrent somatic alterations and uncover likely causal genes^20^. Also, investigating instances where germline variants and somatic alterations jointly affect genes within the same biological pathway may assist in the prioritization of likely causal genes^44^. We anticipate that future studies and the availability of more comprehensive data will help address these limitations.

## Methods

### EOC germline genetics data

In our previous GWAS^9^, we analyzed 25,981 EOC cases and 105,724 controls. The EOC cases were first classified into 2587 mucinous ovarian cancer (MOC) cases and 23,394 nonmucinous ovarian cancer (NMOC) cases. NMOC was further divided into five histotypes, including 15,588 HGSOC cases, 2749 low-grade serous ovarian cancer (LGSOC) cases, 2877 endometrioid ovarian cancer cases, 1427 CCOC cases, and 753 cases of other EOC histotypes (Supplementary Fig. 2). Thirty-three EOC risk regions (Supplementary Data 10) were identified, which were subsequently included in the current study. We noted the definition of risk regions in this study differs from that used in the previous GWAS study. In Dareng et al., risk regions were defined as cytobands, resulting in the identification of 32 cytobands. While in this study, regions were defined as genomic segments, with 33 distinct risk regions identified. Specifically, in 9q31.1, the risk segments for HGSOC and MOC are different. These loci were defined by extending 500 Kb upstream and downstream from the lead SNP position, resulting in a total region of 1 Mb. Fine mapping within these regions identified 4008 credible causal variants mediating the function of risk alleles.

### PCAWG ovarian cancer somatic mutation data

Somatic mutational signatures were acquired from the PCAWG data portal^34^ for 113 EOC samples. Each tumor has 93 mutational signature scores, including 65 for SBS, 11 for double-base-substitution (DBS), and 17 for small ID. The mutational signatures utilized were version 2, as defined in the COSMIC database. Additionally, matched RNA-seq data for 89 EOC samples were obtained from the PCAWG database^34^ for the mutational signature correlation analysis.

Consensus single nucleotide variants (SNVs) and consensus CNAs were also obtained from the PCAWG portal^34^. Those specific to ovarian cancer datasets were extracted for further analysis, comprising 110 samples with available SNVs data and 101 samples with available CNA segmentation files. In total, we identified 981,675 SNVs across 981,172 unique positions, based on data from 110 samples. The distribution of SNVs across these 981,172 positions is as follows: one position (chr17:7577120-7577120) is mutated and observed in nine samples; two positions (chr8:117337127-117337127 and chr17:7578190-7578190) are mutated and observed in four samples; seven positions are mutated and observed in three samples; 475 positions are mutated and observed in two samples; and 980,687 positions are mutated and only observed in one sample.

### Mutational signature analysis

We used three lists of EOC germline susceptibility genes generated from our previous EOC GWAS studies using different bioinformatic tools (Supplementary Data 1): (1) MultiXcan^24^, which performs multi-tissue TWAS, integrating GWAS summary statistics to identify gene expression-trait associations across different tissues. We utilized MultiXcan to find gene expression associations across histotypes of EOC, and identified 83 germline susceptibility genes^9^, (2) MAGMA^25^, which focuses on gene-level association analysis to identify genes associated with complex traits using SNP-level data. We identified a list of 68 EOC susceptibility genes^26^, and (3) chromMAGMA^26^, which extends MAGMA by incorporating chromatin state data to investigate the role of regulatory regions in genetic associations. We identified 155 risk genes associated with EOC. We then applied linear regression models to investigate the relationship between somatic mutational signatures and the expression of germline susceptibility genes. The linear regression results were subject to filtering with a significance threshold of *p* < 0.05 and subsequently grouped by mutational signature. We reported the number of germline susceptibility genes significantly correlated with each mutational signature.

Random permutation analysis was performed to examine the suitability of the germline susceptibility gene lists generated from MultiXcan, MAGMA, and chromMAGMA. For each gene list (MultiXcan: *n* = 83, MAGMA: *n* = 68, chromMAGMA: *n* = 155), an equivalent number of random genes was sampled from the total RNA-seq data. We then repeated the linear regression analysis 10,000 times and counted the number of random genes significantly associated with mutational signatures. In each iteration, if the number of significantly associated random genes exceeds the number of significantly associated germline genes the counter *N* was incremented. *P*-values were calculated as *N*/10,000. Additionally, the distributions of counts from the 10,000 permutations were visualized using boxplots (Supplementary Fig. 1).

### Comprehensive cell-type agnostic and ovarian cancer related functional annotations

We employed two sets of functional annotations. The first set is a baseline model with 25 cell-type agnostic functional annotations from public datasets for genomic categorization^29^, which includes 3′UTR, 5′UTR, Coding, Intron, and Promoter from UCSC Genome Browser^45,46^. Additionally, conserved regions in mammals^47,48^ and CTCF, promoter flanking, transcribed, transcription start site, enhancer, weak enhancer, repressed^49^ were integrated. Digital genomic footprint and TFBS from ENCODE^45^, as well as histone marks, such as H3K27ac, H3K4me1, H3K4me3, and H3K9ac from Roadmap Epigenomics^50^ were incorporated. Furthermore, FANTOM5 Enhancer^51^, DHSs, DHS peaks, Fetal DHS from ENCODE and Roadmap Epigenomics, and Super-Enhancer^52^ were included into the analysis.

The second set of data are ovarian cancer related chromatin states generated as part of our previous study^9^. The chromatin states were defined from a ChromHMM^53^ model which used ChIP-seq data of four histone markers: H3K27ac, H3K4me1, H3K4me3, CTCF, and RNA-seq profiled from 18 EOC associated cell lines (Supplementary Data 11). These cell lines represent two EOC precursor cell types (fallopian tube secretory epithelial cells and ovarian surface epithelial cells) and five EOC histotypes (HGSOC, LGSOC, CCOC, EEC, and MOC). This dataset has a total of eight chromatin states: active region, active promoter, weak promoter, active enhancer, weak enhancer, transcribed, insulator, and low signal (Supplementary Fig. 3). Regions with low signal chromatin states were excluded from our study, because there were no repressive histone marks in our ChromHMM model, and so there was no way to differentiate between low signal and poised regions.

### Functional enrichment of EOC candidate driver mutations

ActiveDriverWGS served as our discovery tool for identifying potential driver mutations throughout the genome, including noncoding and coding regions^54^. The genomic regions were based on 25 cell-type agnostic functional annotations and seven ovarian cancer related chromatin states. For each annotation, ActiveDriverWGS used PCAWG SNVs as inputs to identify elements that are frequently mutated by somatic mutations. We reported the enrichment by calculating the percentage of elements frequently mutated relevant to the total number of regions annotated with the function of interest. After performing the genome-wide enrichment analyses, we then focused on the 33 EOC risk loci and repeated the same calculations. Heatmaps were utilized for visualization with the detailed parameters and codes provided in an *R* markdown file (see Code Availability).

### EOC CNAs in germline risk regions

GISTIC analysis was used to identify significant regions of amplification or deletion across the genome^30^. This analysis was conducted through the online analysis tool GenePattern using the default setting^55^, with PCAWG segmentation files for ovarian cancer samples used as inputs. Functional enrichment of EOC CNAs were done by calculating the coverage of each functional annotation using bedtools version 2.29.0^56^. Similar to the analyses of somatic mutations, we then refined the enrichment analyses to 33 EOC risk loci. The results were visualized with the ggplot function^57^ in *R* version 4.3.2, with detailed parameters and codes provided in an R markdown file (see Code Availability).

### TFBSs frequently disrupted by germline variants or somatic mutations

We used ActiveDriverWGS to identify if binding sites of a TF in the element are significantly mutated by somatic mutations^54^. We collected a set of 746 TFs from the JASPAR 2020 database^58^. ActiveDriverWGS was performed on 6057 frequently mutated elements, with TFBS providing additional information. For germline variants, we used MotifBreakR^59^ to identify motifs frequently disrupted by the 4008 credible causal variants^9^. Here, we further intersected the TFBSs frequently disrupted by germline variants with 15 frequently mutated regions identified by ActiveDriverWGS.

### Reporting summary

Further information on research design is available in the Nature Portfolio Reporting Summary linked to this article.

## Supplementary information

Supplementary Information

Description of Additional Supplementary Files

Supplementary Data 1-11

Reporting summary

## Data Availability

The somatic data analyzed in this study were obtained from ICGC Data Portal at https://dcc.icgc.org/releases/PCAWG/. The cell-type agnostic general annotations used in this study were obtained from https://alkesgroup.broadinstitute.org/LDSCORE/. The EOC related chromatin states were deposited as tracks in the UCSC Genome Browser at https://genome.ucsc.edu/s/pengp/OvarianCancerRegulatoryAtlas. The TFBSs were downloaded from UCSC Genome Browser JASPAR2022 TFBS hg19 Track at http://expdata.cmmt.ubc.ca/JASPAR/downloads/UCSC_tracks/2022/.

## Code availability

The analytical pipeline has been compiled as an R markdown file and is available at https://github.com/pengpclab/Germline-Somatic-Project.
