# Supplementary material for: Characterizing somatic mutations in ovarian cancer germline risk regions
Source: Commun Biol. 2025 Apr 29;8:676. doi: 10.1038/s42003-025-08072-1 (PMC12041368; doi:10.1038/s42003-025-08072-1)
Supplement: Supplementary file 1 — Supplementary Information [file 42003_2025_8072_MOESM1_ESM.pdf]

Supplementary Information for:

Characterizing somatic mutations in ovarian cancer germline risk regions

Ping-Hung Lai[1], Jonathan P. Tyrer[2], Paul Pharoah[1], Simon A. Gayther[3], Michelle R. Jones[4], Pei-Chen Peng[1†]

[1] Department of Computational and Biomedicine, Cedars-Sinai Medical Center, West Hollywood, CA 90069, USA

[2] CR-UK Department of Oncology, University of Cambridge, Strangeways Research Laboratory, Cambridge CB2 0RE, UK

[3] Center for Inherited Oncogenesis, Department of Medicine, UT Health San Antonio, San Antonio, TX 78229, USA

[4] Center for Bioinformatics and Functional Genomics, Department of Biomedical Sciences, Cedars-Sinai Medical Center, Los Angeles, CA 90048, USA

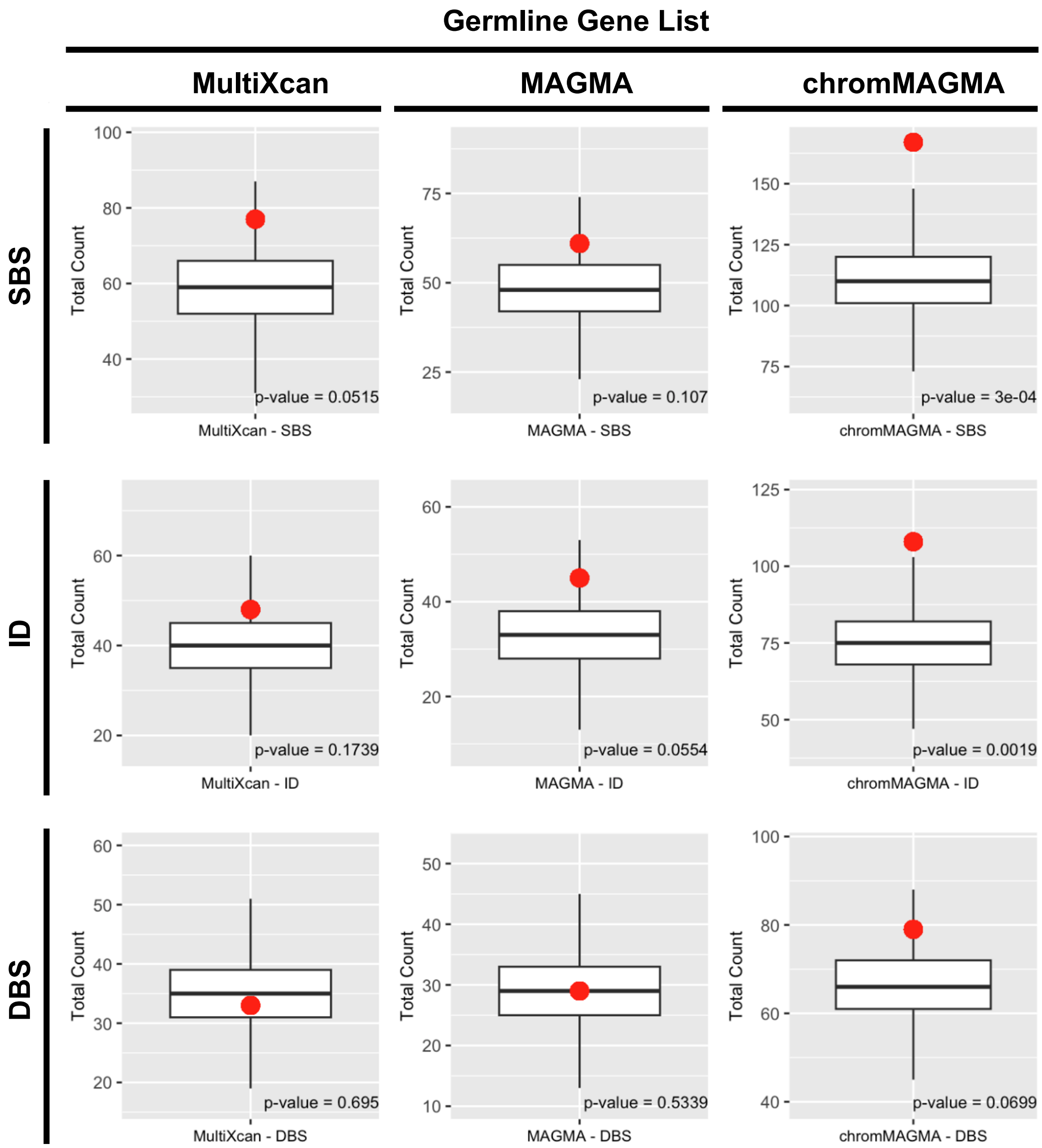

Supplementary Figure 1. Total counts distribution from 10,000 permutation analysis. Boxplots show the distribution of total counts from 10,000 iterations of random permutation analysis, with red dots indicating the initial total counts.

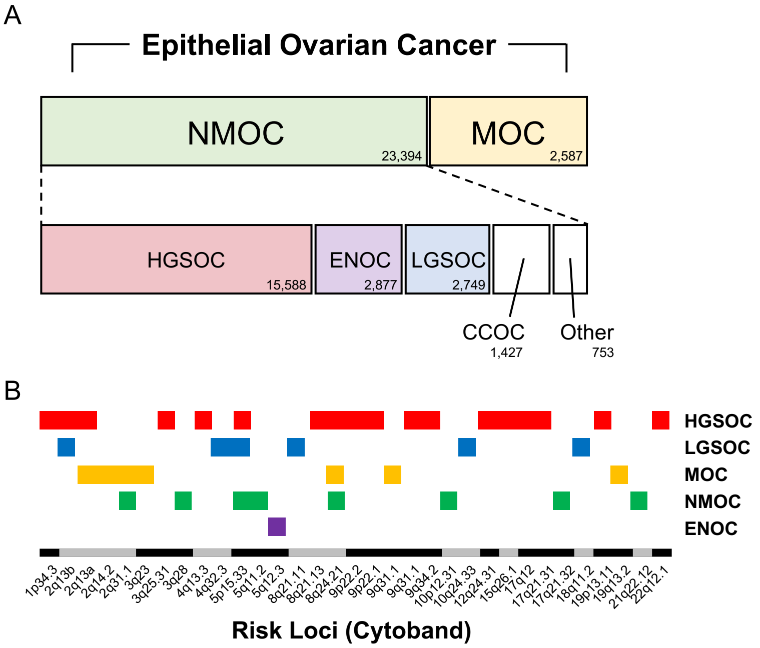

Supplementary Figure 2. Histotypes of Epithelial Ovarian Cancer (EOC) and Associated Genetic Risk Loci. (A) Schematic representation of EOC histotypes. The first categories are Mucinous Ovarian Cancer (MOC) and Non-Mucinous Ovarian Cancer (NMOC). NMOC is further divided into: High-Grade Serous Ovarian Cancer (HGSOC), Endometrioid Ovarian Cancer (EOC), Low-Grade Serous Ovarian Cancer, Clear-Cell Ovarian Cancer (CCOC), and other forms. (B) Distribution of genetic risk loci across EOC histotypes. Colored bars indicate the loci associated with each EOC histotype. Chromosomal locations of each locus are shown along the x-axis, depicted by cytobands.

18 Ovarian Cancer Related Cell Lines

Cell Line	Histotype
JHOC5	CCOC
ES2	CCOC
RMG-II	CCOC
EEC16	EnOC
CaOV3	HGSOC
Hey	HGSOC
UWB1.289	HGSOC
Kuramochi	HGSOC
OAW42	LGSOC
VOA1056	LGSOC
FT246	HGSOC precursor (Fallopian Tube Epithelial)
FT33	HGSOC precursor (Fallopian Tube Epithelial)
FT282	HGSOC precursor (Fallopian Tube Epithelial)
IOSE4	HGSOC precursor (Ovarian Surface Epithelium)
IOSE11	HGSOC precursor (Ovarian Surface Epithelium)
GFTR230	Mucinous OC
MCAS	Mucinous OC
EFO27	Mucinous OC

4 Histone Marks and RNA-seq

Histone Marks
H3K27ac (Active)
H3K4me1 (Enhancer)
H3K4me3 (Promoter)
CTCF
RNA-seq (Transcribed)

ChromHMM.
Ernst & Kellis 2012 Nat Methods.

8 Chromatin States

Chromatin States	
Active Promoter	Active Region
Weak Promoter	Transcribed
Active Enhancer	Insulator
Weak Enhancer	Low Signal

Supplementary Figure 3. Generation of Chromatin States in Ovarian Cancer Cell Lines. The workflow for identifying ovarian cancer-related chromatin states. Eighteen ovarian cancer-related cell lines representing various histotypes, including Clear Cell Ovarian Cancer (CCOC), Endometrioid Ovarian Cancer (EnOC), High-Grade Serous Ovarian Cancer (HGSOC), Low-Grade Serous Ovarian Cancer (LGSOC), and Mucinous Ovarian Cancer (MOC), as well as precursors for HGSOC (Fallopian Tube Epithelium and Ovarian Surface Epithelium). Epigenomic profiling was conducted using four histone marks (H3K27ac, H3K4me1, H3K4me3, and CTCF) along with RNA sequencing (RNA-seq). Chromatin states were then defined using the ChromHMM method (Ernst & Kellis, 2012), integrating these data into eight chromatin states: Active Promoter, Weak Promoter, Active Enhancer, Weak Enhancer, Active Region, Transcribed, Insulator, and Low Signal.
