# Supplementary material for: Characterizing somatic mutations in ovarian cancer germline risk regions
Source: Commun Biol. 2025 Apr 29;8:676. doi: 10.1038/s42003-025-08072-1 (PMC12041368; doi:10.1038/s42003-025-08072-1)
Supplement: Supplementary file 2 — Description of Additional Supplementary Files [file 42003_2025_8072_MOESM2_ESM.pdf]

Description of Additional Supplementary Files

File name: Supplementary Data 1

Description: Gene IDs in three candidate germline susceptibility gene lists

File name: Supplementary Data 2

Description: Mutational signatures and their significantly correlated candidate germline susceptibility genes

File name: Supplementary Data 3

Description: Enrichment analyses of significantly mutated regulatory elements in functional annotations

File name: Supplementary Data 4

Description: Enrichment analyses of significantly mutated regulatory elements in functional annotations, within the EOC risk loci

File name: Supplementary Data 5

Description: GISTIC identified amplified or deleted regions of EOC samples

File name: Supplementary Data 6

Description: GISTIC-identified amplified or deleted regions in EOC samples overlapping with 33 risk loci

File name: Supplementary Data 7

Description: TFBSs frequently disrupted by germline variants in germlinesomatic co-localized regions

File name: Supplementary Data 8

Description: TFBSs frequently disrupted by somatic mutations in germlinesomatic co-localized regions

File name: Supplementary Data 9:

Description: Enrichment analyses of significantly mutated regulatory elements in functional annotations overlapping with PCAWG consortium-identified frequently mutated coding and non-coding regions

File name: Supplementary Data 10

Description: Ovarian cancer germline risk loci

File name: Supplementary Data 11

Description: Epigenomic and transcriptomic profiles of EOC cancer cell lines and precursor cell type
